# Urinary exosome tsRNAs as novel markers for diagnosis and prediction of lupus nephritis

**DOI:** 10.3389/fimmu.2023.1077645

**Published:** 2023-02-09

**Authors:** Shanshan Chen, Xiaoshan Zhang, Kaifang Meng, Yifan Sun, Ruilu Shu, Yan Han, Qingxiu Feng, Zhiyang Li, Ping Yang, Jun Liang

**Affiliations:** ^1^ Department of Rheumatology and Immunology, Affiliated Nanjing Drum Tower Hospital, Medical School of Nanjing University, Nanjing, China; ^2^ Department of Clinical Laboratory, The Affiliated Drum Tower Hospital of Nanjing University Medical School, Nanjing, Jiangsu, China; ^3^ Department of Respiratory and Critical Care Medicine, Nanjing Drum Tower Hospital Clinical College of Nanjing Medical University, Nanjing, Jiangsu, China

**Keywords:** Lupus nephritis, urinary exosomes, tsRNA, non-invasive biomarkers, diagnosis

## Abstract

**Objective:**

Lupus nephritis (LN) is one of the most severe organ manifestations of systemic lupus erythematosus (SLE). Early identification of renal disease in SLE is important. Renal biopsy is currently recognized as the gold standard for diagnosing LN, however, it is invasive and inconvenient for dynamic monitoring. Urine has been considered more promising and valuable than blood in identifying inflamed kidney tissue. Here, we determine whether the signatures of tRNA-derived small noncoding RNA (tsRNA) in urinary exosomes can serve as novel biomarkers for the diagnosis of LN.

**Methods:**

tsRNA sequencing was performed in exosome extracted from pooled urine of 20 LN patients and 20 SLE without LN, and the top 10 upregulated tsRNAs were screened as candidate markers of LN. The candidate urinary exosomal tsRNAs were primarily elected by TaqMan probe-based quantitative reverse transcription-PCR (RT-PCR) in 40 samples (20 LN and 20 SLE without LN) in the training phase. In the validation phase, selected tsRNAs from the training phase were further confirmed in a larger cohort (54 LN patients and 39 SLE without LN). Receiver operating characteristic curve (ROC) analysis was conducted to evaluate the diagnostic efficacy.

**Results:**

Upregulated levels of tRF3-Ile-AAT-1 and tiRNA5-Lys-CTT-1 in the urinary exosomes were observed in LN compared with SLE without LN (*P* < 0.0001 and *P* < 0.001) and healthy controls (*P* < 0.01 and *P* < 0.01), with the area under the curve (AUC) of 0.777 (95% CI: 0.681-0.874, sensitivity 79.63%, specificity 66.69%) and 0.715 (95% CI: 0.610-0.820, sensitivity 66.96%, specificity 76.92%) for discriminating LN from SLE without LN patients. SLE patients with mild activity and moderate to severe activity had higher levels of urinary exosome derived tRF3-Ile AAT-1 (*P* = 0.035 and *P* < 0.001) and tiRNA5-Lys-CTT-1 (*P* = 0.021 and *P* < 0.001) compared with patients with no activity. Moreover, bioinformatics analysis revealed that both of the tsRNAs regulate the immune process by modulating metabolism and signal pathway.

**Conclusion:**

In this study, we demonstrated that urinary exosome tsRNAs can be served as noninvasive biomarkers for the efficient diagnosis and prediction of nephritis in SLE.

## Introduction

Systemic lupus erythematosus (SLE) is an autoimmune disease that predominantly affects women and typically has manifestations in multiple organs. Immune-system aberrations, heritable, hormonal, as well as environmental factors, contribute to the progression of organ damage (1). Lupus nephritis (LN) is a form of glomerulonephritis considered as a severe manifestation of the SLE and continues to be a major cause of morbidity and mortality for SLE patients (2, 3). Routine laboratory examinations such as proteinuria, anti–double-stranded DNA antibodies, and protein-to-creatinine ratio cannot fully reflect disease activity and present with the low diagnostic performance of LN (3–6). To date, renal biopsy was regarded as the gold standard for the diagnosis and classification of scarring and the extent of renal inflammation, but the invasiveness of this approach is not conducive to dynamic monitoring (7). Therefore, it is necessary to search and identify effective non-invasive biomarkers for the detection of LN in SLE.

Exosomes are extracellular vesicles (EVs) secreted by cells mainly consists of phospholipids bilayer structures, with a diameter range from 40 to 160 nm (average ~100 nm), existing in various body fluids including blood, urine and saliva, and plays important roles in remodeling extracellular matrix and transmitting signals and molecules to other cells (8, 9). Endogenous microRNAs (miRNAs), long non coding RNAs (lncRNAs), and tRNA-derived small noncoding RNA (tsRNAs) can be frequently transferred through exosomes from donor cells to recipient cells, displaying important immunomodulatory function in the pathogenesis of various autoimmune diseases, including SLE (10). Recently studies showed that abnormal expression of serum exosomal-miRNAs and lncRNAs was described in LN/SLE patients and both of them are associated with the activity of SLE, histological alterations, and the involvement of renal (11–13). Despite the growing interest in exploring serum exosome derived biomarkers for evaluating activity of disease and predicting the involvement of LN, few biomarkers have been used in clinical practice. Unlike other sample types like tissue or serum, urine testing can be truly non-invasive. Furthermore, the urine is physically close to the site of activity of the renal disease which may be promising specimens for monitoring the patients of SLE.

tsRNAs are novel noncoding small RNAs (14∼40 nt in length), has rarely been described in SLE, including tRNA-derived fragments (tRFs) and tRNA halves (tiRNAs) originate from mature tRNAs or their precursors under the circumstance of enzymatic lysis and stress condition, and have been found stable in the exosome circulating in the biofluids, including urine (11). Furthermore, tsRNAs exert an essential role in the pathophysiology of biological processes by connecting with proteins or mRNA, regulating gene expression, and inhibiting translation (13). Mounting evidence has indicated that the aberrated expression of tsRNAs are deeply implicated in cancer, parasitic disease (12–16). Recently, a few studies including our previous research pay attention to the relationship between SLE and tsRNA (14, 17–19). However, the diagnostic values and biological functions of tsRNAs in urinary exosome, especially for LN, are still ambiguous and intriguing.

In this study, RNA sequencing, qRT-PCR validation, and ROC analysis were performed to identify LN-associated tsRNA signatures in urinary exosome. The urinary exosome derived tsRNA signatures showed enormous potentiality as novel non-invasive biomarkers for diagnosing and predicting LN in SLE.

## Materials and methods

### Subjects and study design

**Figure 1:**
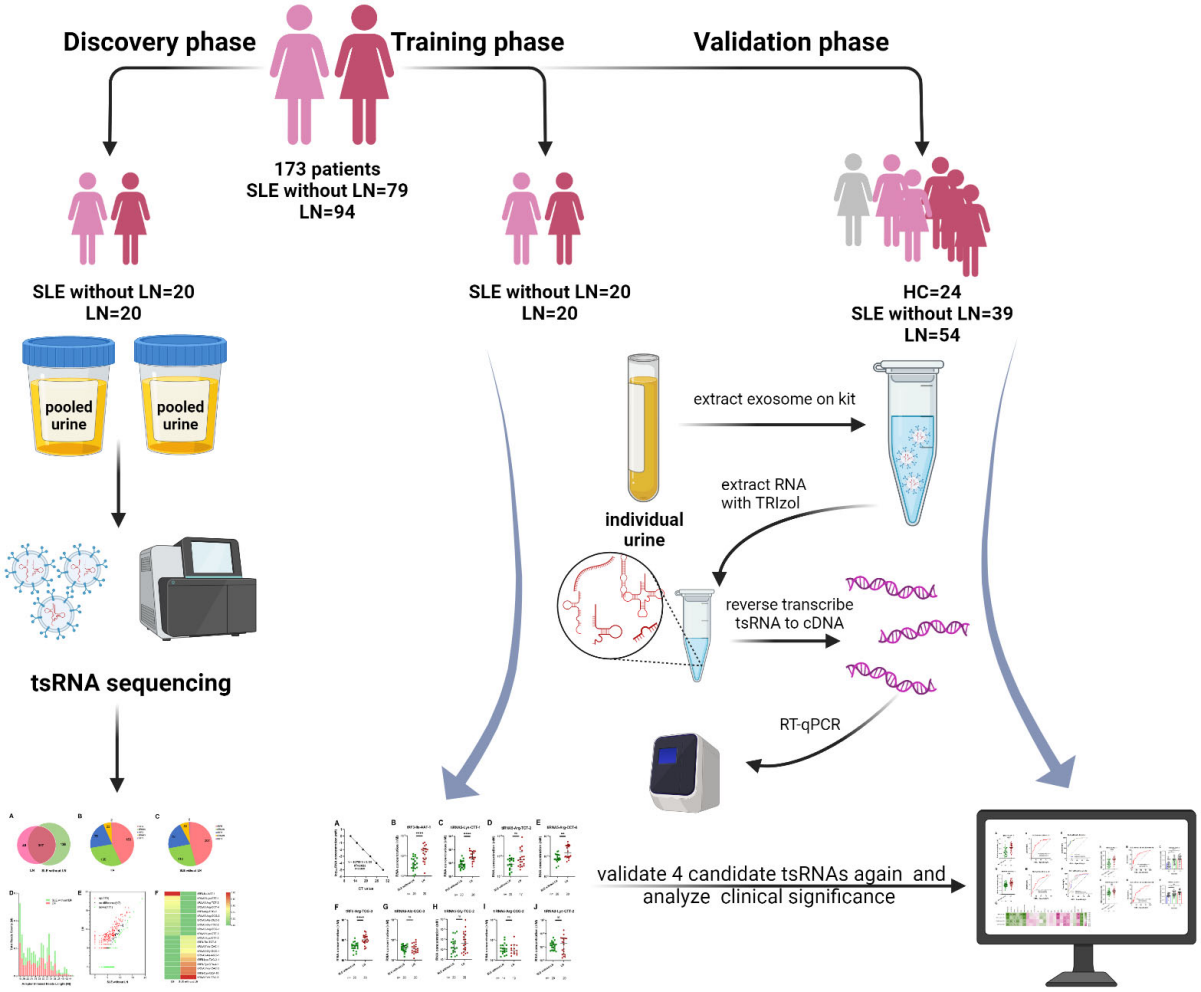
Workflow of the study.

This study including 173 individuals was divided into three phases. In the discovery phase, tsRNA sequencing was conducted in exosomes extracted from pooled urine of 20 lupus nephritis patients (LN) and 20 SLE without LN, and the top 10 upregulated tsRNAs were screened as candidate marker of lupus nephritis. The candidate urinary exosomal tsRNAs were primary elected by qRT-PCR in 40 samples (20 LN and 20 SLE without LN patients) in the training phase. In the validation phase, selected tsRNAs in the training phase were further confirmed in a larger cohort (54 LN and 39 SLE without LN patients), and 24 sex-age matched healthy volunteers from the Center of Physical Examination were enrolled as controls. A total of 94 LN and 79 SLE without LN patients hospitalized in Nanjing Drum Tower Hospital between October 2020 and May 2022 were enrolled in this study (**Figure 1**). The clinical features of both cohorts in validation phase were shown in **Table 1**. The updated criteria of American College of Rheumatology (ACR) 1997 were used to diagnose SLE (20). The activity of disease was evaluated by the systemic lupus erythematosus disease activity index (SLEDAI) (21). Briefly, SLE patients with SLEDAI ≤ 4 were allocated to the stable-disease cohort, SLEDAI vary from 5 to 9 to the mild activity cohort, and SLEDAI ≥ 10 to the moderate-severe activity cohort. All participants provided informed consent and agreed to utilize their urine for research purposes. This study was approved by the ethics committee of Nanjing Drum Tower Hospital and conducted by the principles of the Declaration of Helsinki (1989).

**Table 1:** Statistics of clinical information of validation phase specimens.

clinical characteristic	SLE without LN (n = 39)	LN (n = 54)	*p* value
Age, years	44.0 (32.0-54.0)	39.5(29.0-50.3)	0.286
Male, n (%)	4 (10.25)	5 (9.25)	1
Proteinuria, n (%)	0 (0)	21 (38.89)	<0.001***
Hematuria, n (%)	7 (17.95)	30 (55.56)	<0.001***
pyuria, n (%)	3 (7.69)	18 (33.33)	0.004**
Cylinderuria, n (%)	0 (0)	9 (16.67)	0.007**
24h proteinuria, median (IQR), mg/24h	253.5 (130.8-401.5)	3394.0 (1327.5-5396.5)	<0.001***
ACR, median (IQR), mg/g	32.25 (12.4-104.8)	1811.4 (661.9-4088.2)	<0.001***
WBC, median (IQR), ×10^9/L	4.3 (2.5-5.7)	4.9 (3.6-7.5)	0.015*
Lymphocytes, ×10^9/L	0.80 (0.6-1.2)	0.90 (0.6-1.4)	0.550
Hb, median (IQR), g/L	109.0 (83.0-124.0)	95.0 (70.0-110.0)	0.046*
PLT, median (IQR), ×10^9fL	148.0 (79.0-207.0)	198.0 (131.5-239.3)	0.036*
D-dimer, median (IQR), mg/L	1.2 (0.2-2.0)	1.0 (0.4-1.9)	0.504
ESR, median (IQR), mm/h	28.0 (16.0-59.3)	37.0 (22.0-59.8)	0.211
ALT, median(IQR), U/L	18.5 (12.1 -26.3)	12.1 (8.5-19.0)	0.004**
AST, median (IQR), U/L	18.6 (15.1-26.5)	16.2 (14.0-21.9)	0.042*
blood albumin, median (IQR), g/L	37.4 (34.7-39.0)	31.3 (27.5-34.4)	<0.001***
eGFR, median (IQR), ml/min/1.73m^2	130.5 (97.6-169.9)	90.5 (50.7-117.8)	<0.001***
C3, median (IQR), g/L	0.89 (0.55-1.08)	0.73 (0.49-1.03)	0.208
C4, median (IQR), g/L	0.15 (0.06-0.20)	0.13 (0.06-0.23)	0.694
IgG, median (IQR), IU/mL	13.4 (9.2-20.4)	10.2 (6.9-15.1)	0.017**
Th/Ts, median (IQR)	0.93 (0.65-1.23)	0.47 (0.47-0.97)	0.024*
anti-dsDNA, median (IQR)	67.68 (15.30-229.02)	108.94 (20.24-461.84)	0.260
25-(0H) D3, median (IQR), ng/ mL	16.51 (12.60-21.42)	11.32 (7.32-16.49)	0.001*
SLE-DAI, median (IQR)	4.0 (2.0-6.0)	9.5 (6.0-16.0)	<0.001***
tRF3-Ile-AAT-1, median (IQR)	0.19 (0.07-1.35)	1.88 (0.49-5.73)	<0.001***
tiRNA5-Lys-CTT-1, median (IQR)	0.24 (0.11-0.53)	0.76 (0.29-1.74)	<0.001***

ACR, albumin-to-creatinine ratio; WBC, white blood cells; Hb, hemoglobin; PLT, platelet; ESR, erythrocyte sedimentation rate; eGFR, glomerular filtration rate; C3, complement C3; C4, complement C4; IgG, immunoglobulin G; Th/Ts, helper T cells/suppressor T cells; anti-dsDNA, anti-double stranded DNA antibody; SLE-DAI, systemic lupus erythematosus disease activity index. *p < 0.05, **p < 0.01, and ***p < 0.001 (Mann-Whitney U test).

### RNA sequencing

The 5-adenylated, 3-blocked single-stranded DNA junction is used to connect to the RNAs purified and separated by PAGE electrophoresis cutting glue. One strand cDNA was synthesized by reverse transcription extension with RT primer with unique molecular identifiers (UMI) in the system. High-sensitivity polymerase was used to amplify the cDNA with both 3 ‘and 5’ connectors. Eligible libraries were sequenced on a computer according to the instructions provided by the manufacturer company. A total of 50 cycles of sequencing were performed.

### Isolation and characterization of exosomes

Exosomes were isolated from urine samples with Total Exosome Isolation (from urine) reagent (Thermo Fisher Scientific Baltics UAB) according to the manufacturer’s instructions. First, 180uL urine mixing with 180uL Total Exosome Isolation (from urine) reagent was stored at 4°C for 24 hours. Then the supernatants were discards and the exosome pellets were resuspended in 100 μL 1× phosphate buffer saline (PBS) for further tsRNA extraction. The size and morphology of isolated exosomes were observed by particle size analyzer and transmission electron microscopy. Western blot was used to character exosomes with markers of anti-TSG101 (Abcam, UK), and anti-CD63 (three from Abcam, UK).

### tsRNA extraction and RT-qPCR

Total tsRNAs were extracted from urinary exosomes using the Trizol reagent (Thermo Fisher scientific, US) and were dissolved in water treated by diethylpyrocarbonate (DEPC). The content and purity of acquired tsRNA were examed with OneDrop-2000 (NanoDrop Technologies). tsRNAs were reverse transcribed to cDNA using miRNA 1st Strand cDNA Synthesis Kit (Vazyme Biotech). The quantitative RT-PCR reaction was conducted in 96-well plates with the miRNA Universal SYBR qPCR Master Mix (Vazyme Biotech) in 96-well plates.

### Pathway analysis

To gain insight into the function of the target genes of tsRNAs, we implemented gene ontology (GO), annotations Kyoto Encyclopedia of Genes and Genomes (KEGG) and wiki pathway enrichment analysis using the tRF target gene database (http://www.rnanut.net/tRFTar/). Significance was established on *P*-value (*P* < 0.05) and Q-values.

### Statistical analysis

Continuous variables were presented as the median (interquartile range [IQR]) according to the normality test, and the differences between the groups were determined by the Mann-Whitney U test. Spearman rank test were employed to analyze the correlations between 4 tsRNAs and clinical variables. Receiver operator characteristic (ROC) analyses of variables were performed to calculate the areas under the ROC curves (AUCs) for identifying LN from SLE without LN. Statistical analyses were performed using GraphPad Prism version 8 (GraphPad Software Inc., La Jolla, CA, USA). A two-sided *P* less than 0.05 was considered statistically significant.

### Data and materials availability

The raw data obtained from tsRNA sequencing have been deposited in GEO under registration code PRJNA890378. The primary discoveries of this study were summarized in the manuscript and raw data that support the results of this study are available from the corresponding author upon reasonable request.

## Results

### Characteristics of urinary exosomes

**Figure 2:**
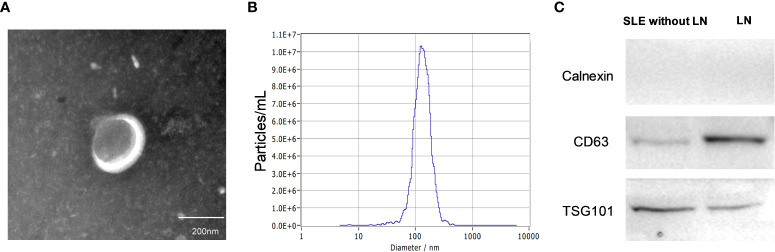
Characteristics of urinary exosomes. **(A, B)** Transmission electron micrograph and nanoparticle tracking analysis confirmed that the isolated small vesicles are urinary exosomes (with vesicle of 129 nm average diameters). **(C)** Western blotting showed the biomarkers of exosomes such as CD63, and TSG101.

The urinary exosomes characterized by cup-shaped morphology with vesicles of 129 nm average diameters were examined using transmission electron microscopy (TEM) and nanoparticle tracking analysis (NTA) (**Figures 2A, B**). The presence of exosomes markers including CD63, and TSG101 (9) were verified by Western blot analysis (**Figure 2C**). Collectively, these results demonstrated the existence of exosomes in urine, which laid the foundation for further research on exosome biomarkers.

### Ectopic urinary exosomes derived tsRNAs profiles in LN patients

**Figure 3:**
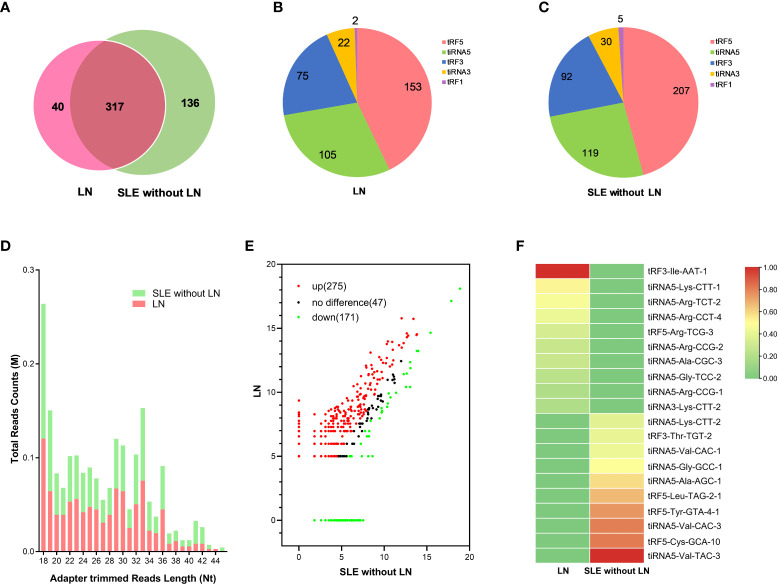
Analysis of differentially expressed tsRNAs in UEs of LN patients. **(A)** Venn diagram of urinary exosomes derived tsRNAs in LNs and SLE without LN patients. **(B, C)** The distribution of 5 types urinary exosomes derived tsRNAs in the two cohorts. **(D)** The profiles of various length of tsRNAs in urinary exosomes between the two cohorts. **(E)** Scatter plots of differentially expressed tsRNAs. Red and green dots indicated upregulated and downregulated tsRNAs (log2 fold-change > 1.5 between the two compared cohorts), and black dots indicated non-differentially expressed tsRNAs. **(F)** Hierarchical clustering indicated the profiles of top 10 upregulated and downregulated tsRNAs between two cohorts.

To identify the tsRNAs profile in the urinary exosomes of LN patients, all participates were divided into two cohorts based on the clinical classification criteria, including LN and SLE without LN patients. Urine pooled from 20 LN patients and 20 SLE without LN patients were utilized to extract exosomes, followed by tsRNA sequencing (GEO number: PRJNA890378). The amount and classification of tsRNAs in urinary exosomes were described by Venn diagram and pie chart in both LN and SLE without LN (**Figures 3A–C**). Length distribution of tsRNAs was further analyzed and various number of bases of tsRNA exhibited significant bias between the two groups (**Figure 3D**). In scatter plot analysis, 275 upregulated and 171 downregulated tsRNAs, which satisfied the criterion that log_2_ fold-change > 1.5 by sequencing detection, were observed in LN compared with SLE without LN patients (**Figure 3**). The top 10 upregulated tsRNAs visualized using hierarchical clustering were selected as candidate markers of lupus nephritis (**Figure 3F**).

### Identification of differentially expressed urinary exosomes derived tsRNAs in LN and SLE without LN patients

**Figure 4:**
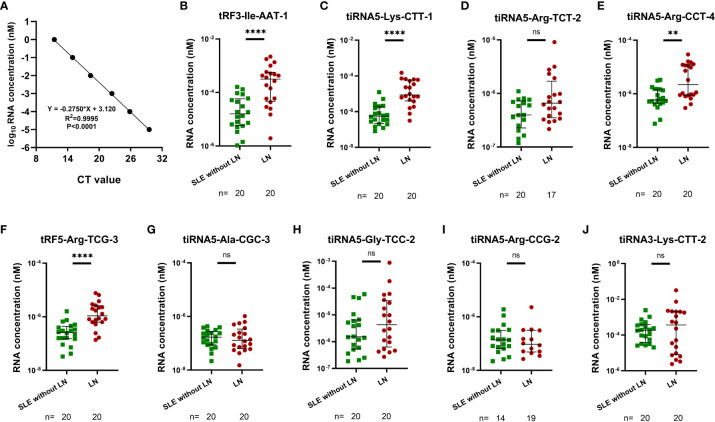
Identification of differentially expressed urinary exosomes derived tsRNAs in LNs and SLE without LN patients. **(A)** Linear standard curve of urinary exosomes derived tsRNAs concentration. **(B–J)** Differential expression of 9 tsRNAs verified by RT-qPCR in LN and SLE without LN patients. tRF3-Ile-AAT-1, tiRNA5-Lys-CTT-1, tiRNA5-Arg-CCT-4 and tRF5-Arg-TCG-3 were significantly upregulated in LNs compared with SLE without LN patients. P value of Mann Whitney U test: (**P < 0.01, ****P < 0.0001).

RT-qPCR assay was performed to validate the sequencing results of urinary exosomes from 20 LN and 20 SLE without LN patients in the training phase. The absolute quantification for different expression of tsRNAs was determined by the linear standard curve from 10 f-mol/L to 1 nmol/L (**Figure 4A**). Specific primers were designed for the 10 candidate tsRNAs and the tsRNAs was successfully amplified except of tiRNA5-Arg-CCG-2, which could not be amplified and was thus eliminated from this study. Elevated expression of tRF3-Ile-AAT-1, tiRNA5-Lys-CTT-1, tiRNA5-Arg-CCT-4, and tRF5-Arg-TCG-3 were measured in the urinary exosomes of LN compared with SLE without LN patients (**Figures 4B–J**), implying urinary exosomes derived tsRNAs may serve as promising biomarkers for the diagnosis of LN.

### Diagnostic value of urinary exosomes derived tsRNAs in the validation phase

**Figure 5:**
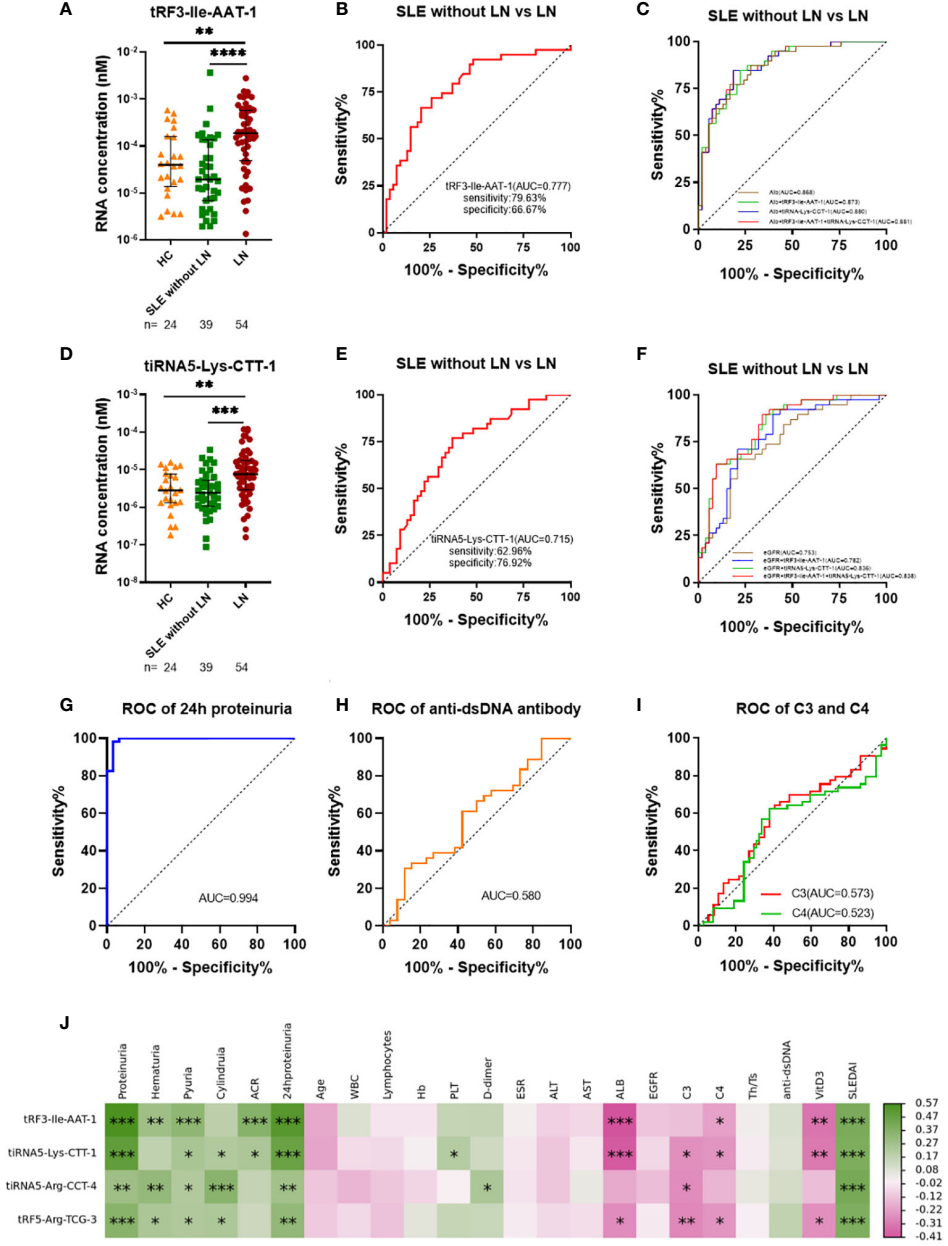
Diagnostic value of urinary exosomes derived tsRNAs in the validated stage. **(A)** Overexpression of tRF3-Ile AAT-1 in LNs compared with SLE without LN patients and healthy controls. **(B)** Receiver operator characteristic (ROC) curve of tRF3-Ile AAT-1 in distinguishing LNs from SLE without LN patients. **(C)** ROC combined diagnostic analyses of tRF3-Ile AAT-1and Alb, tiRNA5-Lys-CTT-1 and Alb, as well as tRF3-Ile AAT-1, tiRNA5-Lys-CTT-1 and Alb in discriminating LNs from SLE without LN patients.**(D)** Overexpression of tiRNA5-Lys-CTT-1 in LNs compared with SLE without LN patients and healthy controls. **(E)** ROC curve of tiRNA5-Lys-CTT-1 in distinguishing LNs from SLE without LN patients. **(F)** ROC combined diagnostic analyses of tRF3-Ile AAT-1and eGFR, tiRNA5-Lys-CTT-1 and eGFR, as well as tRF3-Ile AAT-1, tiRNA5-Lys-CTT-1 and eGFR in discriminating LNs from SLE without LN patients. **(G)** ROC curve of 24h proteinuria in distinguishing LNs from SLE without LN patients. **(H)** ROC curve of anti-dsDNA in distinguishing LNs from SLE without LN patients. **(I)** ROC curves of complement C3 and C4 in distinguishing LNs from SLE without LN patients. **(J)** Correlation analyses between tsRNAs and clinical variables. Green and red color represent the positive correlation and negative correlation, and the depth of the color represents the degree of correlation. (*P < 0.05, **P < 0.01, ***P < 0.001, ****P < 0.0001).

We enrolled 54 LN, 39 SLE without LN patients and 24 healthy controls in the validation phase, in which tRF3-Ile-AAT-1 and tiRNA5-Lys-CTT-1 were remarkably increased in LN compared with SLE without LN patients (*P* < 0.0001 and *P* < 0.001) and healthy controls (*P* < 0.01 and *P* < 0.01) (**Figures 5A, D**), whereas tiRNA5-Arg-CCT-4 and tRF5-Arg-TCG-3 showed no difference among the three cohorts. The AUCs of tRF3-Ile-AAT-1 and tiRNA5-Lys-CTT-1 were 0.777 (95% confidence interval [CI]: 0.681-0.874, sensitivity 79.63%, specificity 66.69%) and 0.715 (95% CI: 0.610-0.820, sensitivity 66.96%, specificity 76.92%) for discriminating LN from SLE without LN patients (**Figures 5B, E**). In addition, 24-hour proteinuria showed good diagnostic value in identifying nephritis in SLE patients (AUC=0.94), whereas anti-dsDNA, complement C3 and C4 have limited diagnostic value in lupus nephritis (**Figures 5G–I**). To further demonstrate the clinical significance of tRF3-Ile-AAT-1 and tiRNA5-Lys-CTT-1 expression in SLE patients, we investigated the association between the tsRNAs levels and various clinical parameters in a total of 93 SLE patients. The results indicated that urinary exosomes derived tRF3-Ile-AAT-1 and tiRNA5-Lys-CTT-1 were positively correlated with ACR, SLEDAI and 24-hour proteinuria while negatively associated with albumin (Alb), complement C3 and Vitamin D3 (**Figure 5J**). Then we explored the predictive accuracy of the tRF3-Ile-AAT-1 and tiRNA5-Lys-CTT-1 combined with clinical indicators. The AUCs of tRF3-Ile-AAT-1 and Alb as well as tiRNA5-Lys-CTT-1 and Alb were 0.873 (95% CI: 0.802–0.944) and 0.880 (95% CI: 0.811–0.949) between individuals of LN and SLE without LN patients. As expected, integrated panel of tRF3-Ile-AAT-1, tiRNA5-Lys-CTT-1 and Alb resulted in the highest AUC of 0.881 (95% CI: 0.813-0.950, sensitivity 83.72%, specificity 94.19%) (**Figure 5C**). The AUCs of tRF3-Ile-AAT-1 and eGFR as well as tiRNA5-Lys-CTT-1 and eGFR were 0.783 (95% CI: 0.687-0.878) and 0.836 (95% CI: 0.754-0.917). Similarly, integrated panel of tRF3-Ile-AAT-1, tiRNA5-Lys-CTT-1 and eGFR resulted in the highest AUC of 0.838 (95% CI: 0.757-0.918, sensitivity 83.72%, specificity 94.19%) (**Figure 5F**).

### Significance of tRF3-Ile-AAT-1 and tiRNA5-Lys-CTT-1 in clinical practice

**Figure 6:**
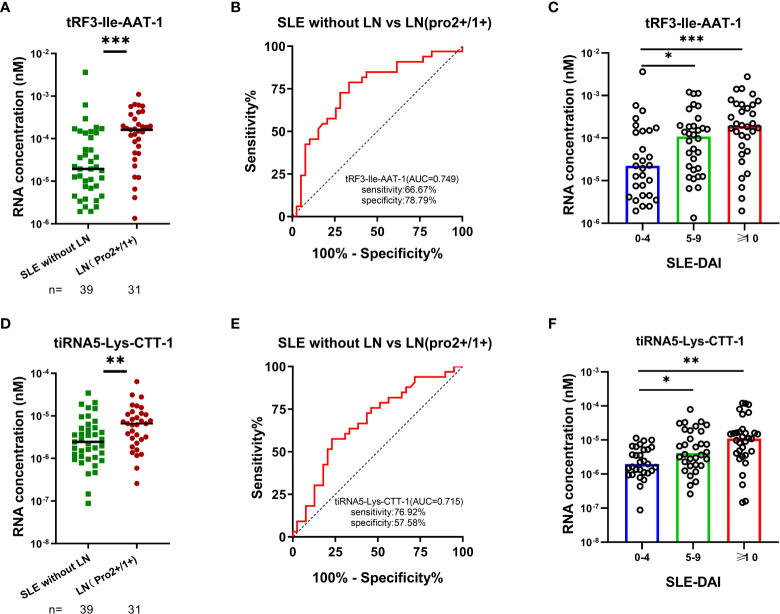
Significance of tRF3-Ile AAT-1 and tiRNA5-Lys-CTT-1 in clinical practice. **(A, B)** Diagnostic value of tRF3-Ile AAT-1 in LNs with proteinuria (2+)/ (1+) and SLE without LN patients. **(D, E)** Diagnostic value of tiRNA5-Lys-CTT-1 in LNs with proteinuria (2+)/ (1+) and SLE without LN patients. **(C, F)** Correlation between tRF3-Ile AAT-1, tiRNA5-Lys-CTT-1 and the activity of SLE measured by SLE-DAI 2000 (0-4, no activity; 5-9, mild activity; ≥10, moderate to severe activity).

Routine urinalysis usually utilized as indicators for monitoring the occurrence and progression of LN with a high specificity but low sensitivity. LN with weakly positive proteinuria (2+/1+) are commonly ignored and resulted in a delay of diagnosis in clinical practice. Elevated tRF3-Ile AAT-1 levels were observed in LN with proteinuria (2+)/(1+) compared with SLE without LN patients (*P* < 0.001), and the AUC of tRF3-Ile-AAT-1 were 0.749 (95% CI: 0.633-0.865, sensitivity 66.67%, specificity 78.79%) for discriminating LN with proteinuria (2+)/(1+) from SLE without LN patients (**Figures 6A, B**). Similarly, tiRNA5-Lys-CTT-1 levels were higher in LN with proteinuria (2+)/(1+) compared with SLE without LN patients (*P* = 0.009), and the AUC was 0.715 (95% CI: 0.554-0.804, sensitivity 76.92%, specificity 57.58%) (**Figures 6D, E**). Intriguingly, SLE patients with mild activity and moderate to severe activity had higher urinary exosomes derived tRF3-Ile AAT-1 (*P* = 0.035 and *P* < 0.001) and tiRNA5-Lys-CTT-1 (*P* = 0.021 and *P* < 0.001) levels compared with patients with no activity, whereas there was no difference of tRF3-Ile-AAT-1 and tiRNA5-Lys-CTT-1 between SLE patients with mild activity and moderate to severe activity (both *P* > 0.05) (**Figures 6C, F**).

### Enrichment analysis of tRF3-Ile AAT-1 and tiRNA5-Lys-CTT-1

**Figure 7:**
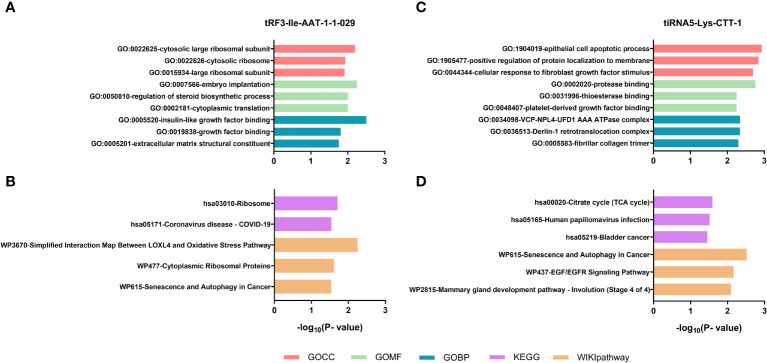
Enrichment analysis of tRF3-Ile AAT-1 and tiRNA5-Lys-CTT-1. GO terms analysis of tRF3-Ile AAT-1 **(A)**. Signal pathway analysis of tRF3-Ile AAT-1 **(B)**. GO terms analysis of tiRNA5-Lys-CTT-1 **(C)**. Signal pathway analysis of tiRNA5-Lys-CTT-1 **(D)**.

To investigate the potential functions of tRF3-Ile AAT-1 and tiRNA5-Lys-CTT-1, we performed gene ontology analyses including cellular components, biological processes and molecular functions as well as signal pathway analyses containing KEGG and wiki pathways (*P* < 0.05). The GO project of tRF3-Ile AAT-1 were enriched in regulation of steroid biosynthetic process, embryo implantation, insulin-like growth factor binding, growth factor binding and so on (**Figure 7A**). Surprisingly, the KEGG showed that there may be some correlation between tRF3-Ile AAT-1 and coronavirus disease - COVID-19 (**Figure 7B**). GO terms targeted by tiRNA5-Lys-CTT-1 including cellular response to fibroblast growth factor stimulus, VCP-NPL4-UFD1 AAA ATPase complex, platelet-derived growth factor binding, and other GO stems was found to be enrich (**Figure 7C**). Signal pathway analysis of tiRNA5-Lys-CTT-1 showed the tsRNA was involved in the pathway of Human papillomavirus infection, Senescence and Autophagy in Cancer, EGF/EGFR Signaling Pathway (**Figure 7D**). Hence, these results remind us of the tsRNAs were likely to participate in the pathogenesis of lupus *via* affecting these functions.

## Discussion

SLE is an autoimmune disease characterized by abnormal activation of lymphocytes and abnormal accumulation of autoantibodies (22, 23). Lupus nephritis is one of the most devastating manifestations of SLE, during which 40% of them will develop chronic kidney disease and 10–20% of them will progress to end-stage renal disease within 5 years after diagnosis (14, 24). Thus, early detection and prediction of the progression of LN is critical for the management and treatment of SLE. As the main diagnostic criterion for LN, the 24-hour urinary protein provides good estimate of proteinuria. However, this method still has many drawbacks except for the inconvenience and the length time taken to undertake the test, such as inaccurate timing, partial urine sample loss during urine retention, poor patient compliance for urine protein test, physical activity, and not appropriate for outpatients or children (14, 24–28). Similarly, the usefulness of examining the urinary sediment has been the subject of debate because of the huge variation in quantifying the finding (3). In fact, protein-to-creatinine ratio in spot urine has replaced 24-hour urinary protein collections in many centers, while this approach has not been carefully validated (3). In addition, non-conventional biomarkers, like monocyte chemoattractant protein-1 (MCP-1) (29, 30), kidney injury molecule–1 (KIM-1) (30), vascular cell adhesion molecule-1 (VCAM-1) (31), neutrophil gelatinase-associated lipocalin (NGAL) (32), and microRNAs (33) have been described in the diagnosis and disease activity monitoring of LN, while the specificity and sensitivity are typically difficult to meet clinical practice standards. Therefore, reliable non-invasive biomarkers with higher sensitivity and specificity for the early detection and risk stratification of LN are urgently needed.

Urinary exosomes originated from glomerular podocytes or renal tubules epithelial cells enclose various species of RNA including tsRNAs (34, 35). Zhu et al. (36) found that the bladder, endothelial cell, basal cell, monocyte, and dendritic cell may closely participate in the formation of urine exosomes by investigating the genetic sources of urinary exosomes both at the levels of organs and cells. The evidence showed that the existence of CD47 on exosomes prolongs their half-life period in the circulation (37). In addtion, the tsRNAs were encapsulated in exosomes protecting them from degradation, and have a good deal of methylated modifications and terminal modifications making them stable in body fluids (38).The changes of urinary exosomal tsRNAs may reflect renal injury so that can be expected to be a potential clinical biomarker without need for an invasive tissue biopsy.

Recently, Park. Et al. reported that tsRNAs were capable of regulating the chromatin states in immune cells, indicating that tsRNAs involved critically in the occurrence and development of immune-related diseases (11). Chioyu et al. has described that tsRNA were enrich in exosomes released by T cells, which played a critical role in the organ involvement of SLE (39). More importantly, the content of tsRNAs in vesicles were significantly higher than miRNAs (39–41). tsRNAs in serum as biomarkers of SLE and LN patients were confirmed in our previous studies (14, 15).Based on the perspective of non-invasive liquid biopsy, this was the first report to explore whether urinary exosome derived tsRNA signatures could be considered as biomarkers for distinguishing LN from SLE patients

In our study, exosomes were characterized by TEM and NTA and double phospholipid bilayer cup-shaped vesicle structures with average particle about 129 nm were obtained, which was consistent with previous study (42). According to the sequencing results, we discovered 275 upregulated urinary exosomes derived tsRNAs in LN compared with SLE without LN patients. In training phase, 10 tsRNAs were verified increasing expression in LN. In validation phase, based on more clinical specimens, we further narrowed the candidates tsRNAs down to tRF3-Ile AAT-1 and tiRNA5-Lys-CTT-1. In addition, ROC curve analyses showed the high-precision discriminatory power of tRF3-Ile AAT-1 and tiRNA5-Lys-CTT-1 between SLE patients with and without LN. The evidence showed that angiogenin cleaves t-RNA into fragment as tsRNAs under conditions of cell stress, which contributes to immune system dysregulation, autoantibody production and fatal comorbidities of SLE patients (39, 40). tRFs and tiRNAs inhibit translation in a variety of ways (38), and possibly slow down the progression of some inflammatory reaction manifestation of SLE in the kidneys. In our study, tRF3-Ile-AAT-1 and tiRNA5-Lys-CTT-1 showed good diagnostic value in identifying LN in SLE patients, which are better than conventional biomarkers such as anti-dsDNA antibody, C3 and C4. We further created a panel consisted of both tsRNAs and clinical parameters such as Alb and EGFR, demonstrating higher diagnostic value in distinguishing LN from SLE patients. SLE-DAI is a reliable indicator reflecting SLE activity scored by clinical symptoms and auxiliary examination, while 24 hours proteinuria is considered as the main diagnostic criteria for LN contraindicated to renal biopsy (3, 21). The two tsRNAs were positively correlated with proteinuria and SLE-DAI score, implying that tsRNAs may be involved in the activity of SLE and progression of LN. In addition, LN often present with mild proteinuria (3), and our study analyzed the significance of tsRNAs in clinical practice and found that it could identify active LN who were often missed because the urine protein in routine examination did not reach strong positive. Besides, both tsRNAs in our study can distinguish active SLE from stable SLE patients, which can help rheumatologist judge the severity and risk of disease. Intriguingly, there was a strong correlation between the two tsRNAs, while mechanism of exactly how they cooperate in LN depends on further research. GO analysis showed tRF3-Ile AAT-1 was enriched in insulin-like growth factor binding, which influenced autoimmune by modulating signaling pathways relevant to Th17/Treg balance (42). tiRNA5-Lys-CTT-1 was found involved with platelet-derived growth factor (PDGF) binding, there were studies showed that PDGF family have been concerned as an autoantigen target in the serum of SLE patients and play role in the pathogenesis of renal fibrosis (43). On the strength of above, we can hypothesize that tsRNAs may be involved in the occurrence and development of LN by participating in these pathways.

In summary, we first identified novel urine exosome derived two-tsRNAs signature in SLE patients. We then demonstrated that urine exosome derived tRF3-Ile AAT-1, tiRNA5-Lys-CTT-1 could be used as novel promising biomarkers for distinguishing LN from SLE patients. Furthermore, our study also revealed the potential biological functions of the two novel urine exosome derived tsRNAs. However, because of the limited samples and single-center experience of our study, randomized clinical trials will be needed to evaluate the possible application of the two-tsRNAs signature in the diagnosis and prognosis of LN in the future.

## Data availability statement

The datasets presented in this study can be found in online repositories. The names of the repository/repositories and accession number(s) can be found in the article/**Supplementary Material**.

## Ethics statement

The studies involving human participants were reviewed and approved by Ethics Committee of the Affiliated Drum Tower Hospital of Nanjing University Medical School. The patients/participants provided their written informed consent to participate in this study. Written informed consent was obtained from the individual(s) for the publication of any potentially identifiable images or data included in this article.

## Author contributions

SC and XZ contributed equally to this work. SC and PY writing the original draft. XZ: methodology and data validation. ZL, JL: conceptualization and supervision. PY is the chief designer of the whole experiment. All authors contributed to the article and approved the submitted version.
