# Supplementary material for: Urinary exosome tsRNAs as novel markers for diagnosis and prediction of lupus nephritis
Source: Front Immunol. 2023 Feb 9;14:1077645. doi: 10.3389/fimmu.2023.1077645 (PMC9946979; doi:10.3389/fimmu.2023.1077645)
Supplement: Supplementary file 1 [file DataSheet_1.docx]

**Supplementary Materials:**

**Supplementary Table 1. Statistics of clinical information of discovery phase specimens.**

| **clinical characteristic** | **SLE without LN**  **(n = 20)** | **LN (n = 20)** | ***p* value** |
| --- | --- | --- | --- |
| Age, years | 29.0 (22.0-36.0) | 31 (25.0-33.0) | 0.803 |
| Male, n (%) | 2 (10.0) | 2 (10.0) | 1 |
| Proteinuria, n (%) | 0 (0) | 16 (80.0) | <0.001^***^ |
| Hematuria, n (%) | 1 (5.0) | 14 (70.0) | <0.001^***^ |
| pyuria, n (%) | 0 (0) | 8 (40.0) | <0.001^***^ |
| Cylinderuria, n (%) | 0 (0) | 3 (15.0) | <0.001^***^ |
| 24h proteinuria, median (IQR), mg/24h | 250.0 (91.7-340.0) | 2472.5.0 (1287.0-5746.0) | <0.001^***^ |
| ACR, median (IQR), mg/g | 24.05 (10.8-74.8) | 1914.5 (636.1-3564.7) | <0.001^***^ |
| WBC, median (IQR), ×10^9/L | 3.8 (2.9-5.2) | 5.6 (4.3-8.4) | 0.007^**^ |
| Lymphocytes, ×10^9/L | 0.8 (0.5-1.0) | 0.90 (0.6-1.9) | 0.253 |
| Hb, median (IQR), g/L | 109.0 (86.3-128.0) | 79.0 (64.0-107.0) | 0.033^*^ |
| PLT, median (IQR), ×10^9/L | 161.0 (90.5-216.0) | 195.0 (120.0-272.3) | 0.317 |
| D-dimer, median (IQR), mg/L | 1.2 (0.4-2.2) | 1.3 (0.5-1.8) | 0.844 |
| ESR, median (IQR), mm/h | 24.0 (16.0-42.5) | 32.0 (22.3-55.5) | 0.244 |
| ALT, median (IQR), U/L | 16.5 (10.9 -20.7) | 11.9 (8.5-16.5) | 0.064 |
| AST, median (IQR), U/L | 16.4 (14.2-26.0) | 15.9 (14.1-20.8) | 0.561 |
| blood albumin, median (IQR), g/L | 38.2 (37.1-39.8) | 31.5 (25.8-36.0) | <0.001^***^ |
| eGFR, median (IQR), ml/min/1.73m^2 | 137.3 (97.9-173.2) | 80.3 (29.2-102.8) | <0.001^***^ |
| C3, median (IQR), g/L | 0.92 (0.68-1.19) | 0.74 (0.52-1.04) | 0.086 |
| C4, median (IQR), g/L | 0.15 (0.11-0.22) | 0.12 (0.06-0.14) | 0.190 |
| IgG, median (IQR), IU/mL | 13.2 (9.3-19.8) | 8.4 (5.9-11.3) | 0.007^**^ |
| Th/Ts, median (IQR) | 0.94 (0.68-1.24) | 0.93(0.47-1.07) | 0.288 |
| anti-dsDNA, median (IQR) | 63.8(34.5-256.2) | 86.9 (16.3-767.0) | 0.794 |
| 25-(OH) D3, median (IQR), ng/mL | 16.9 (10.0-23.0) | 11.6 (7.3-16.8) | 0.060 |
| SLE-DAI, median (IQR) | 4.0 (3.0-7.0) | 11.5 (8.0-16.0) | <0.001^***^ |

Abbreviations: ACR, albumin-to-creatinine ratio; WBC, white blood cells; Hb, hemoglobin; PLT, platelet; ESR, erythrocyte sedimentation rate; eGFR, glomerular filtration rate; C3, complement C3; C4, complement C4; IgG, immunoglobulin G; Th/Ts, helper T cells/suppressor T cells; anti-dsDNA, anti-double stranded DNA antibody; SLE-DAI, systemic lupus erythematosus disease activity index. **p < 0.05, **p < 0.01, and ***p < 0.001* (Mann-Whitney U test).

**Supplementary Figure 1**


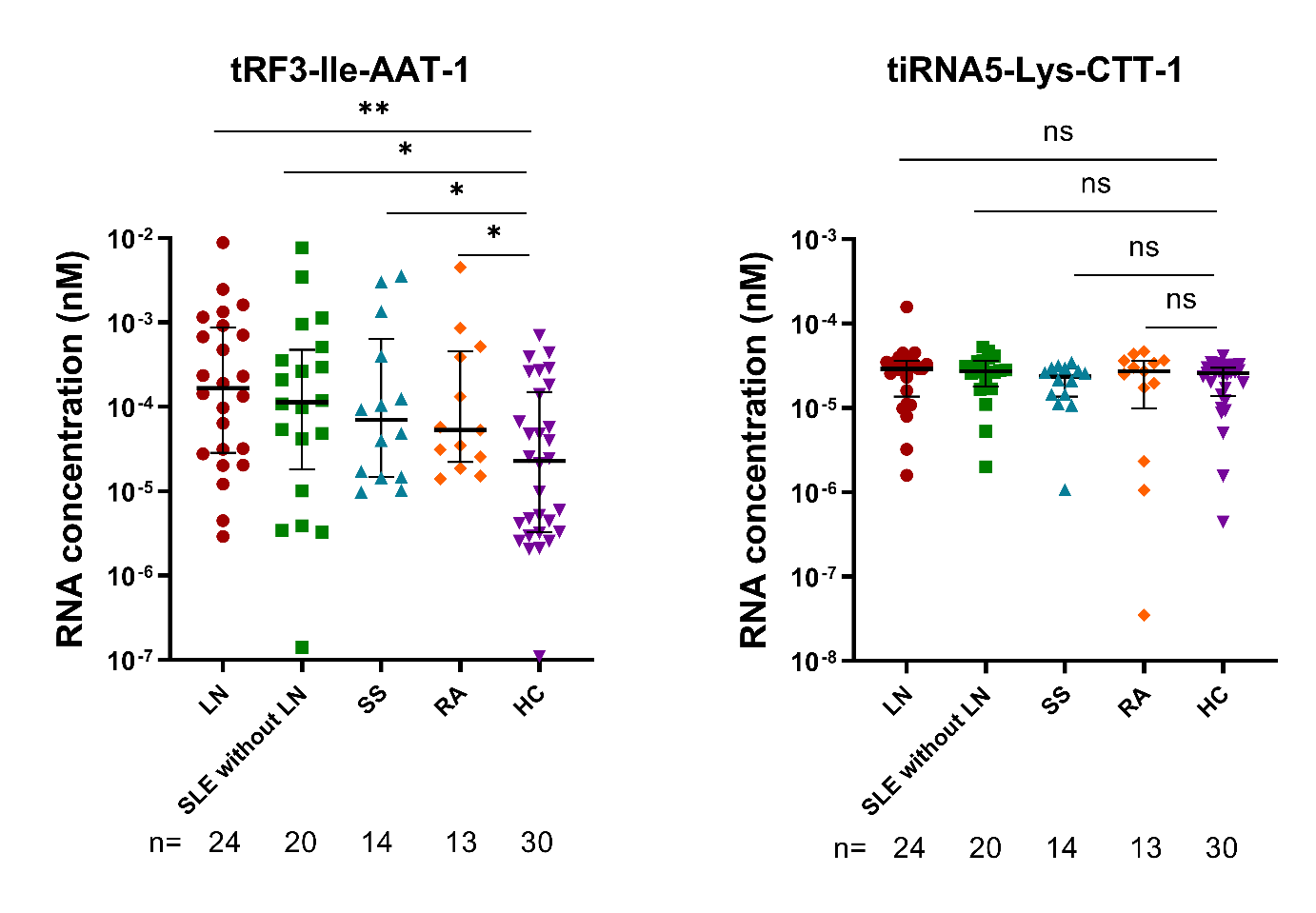


SS, Sjogren's syndrome; RA, rheumatoid arthritis; HC, healthy controls.

Supplementary Figure 1: The expression of tRF3-Ile-AAT-1 and tiRNA5-Lys-CTT-1 in different immune diseases and healthy controls.
